# From Microbial Switches to Metabolic Sensors: Rewiring the Gut–Brain Kynurenine Circuit

**DOI:** 10.3390/biomedicines13082020

**Published:** 2025-08-19

**Authors:** Masaru Tanaka, László Vécsei

**Affiliations:** 1 Danube Neuroscience Research Laboratory, HUN-REN-SZTE Neuroscience Research Group, Hungarian Research Network, University of Szeged, Tisza Lajos krt. 113, H-6725 Szeged, Hungary; 2 Department of Neurology, Albert Szent-Györgyi Medical School, University of Szeged, Semmelweis u. 6, H-6725 Szeged, Hungary

**Keywords:** tryptophan, kynurenine, indoleamine 2,3-dioxygenase, tryptophan 2,3-dioxygenase, microbiotal brain–gut axis, circadian rhythm, chronotherapy, probiotics, precision medicine

## Abstract

The kynurenine (KYN) metabolic pathway sits at the crossroads of immunity, metabolism, and neurobiology, yet its clinical translation remains fragmented. Emerging spatial omics, wearable chronobiology, and synthetic microbiota studies reveal that tryptophan (Trp) metabolism is regulated by distinct cellular “checkpoints” along the gut–brain axis, finely modulated by sex differences, circadian rhythms, and microbiome composition. However, current interventions tackle single levers in isolation, leaving a key gap in the precision control of Trp’s fate. To address this, we drew upon an extensive body of the primary literature and databases, mapping enzyme expression across tissues at single-cell resolution and linking these profiles to clinical trials investigating dual indoleamine 2,3-dioxygenase 1 (IDO1)/tryptophan 2,3-dioxygenase (TDO) inhibitors, engineered probiotics, and chrono-modulated dosing strategies. We then developed decision-tree algorithms that rank therapeutic combinations against biomarker feedback loops derived from real-time saliva, plasma, and stool metabolomics. This synthesis pinpoints microglial and endothelial KYN hotspots, quantifies sex-specific chronotherapeutic windows, and identifies engineered Bifidobacterium consortia and dual inhibitors as synergistic nodes capable of reducing immunosuppressive KYN while preserving neuroprotective kynurenic acid. Here, we highlight a framework that couples lifestyle levers, bio-engineered microbes, and adaptive pharmaco-regimens into closed-loop “smart protocols.” By charting these intersections, this study offers a roadmap for biomarker-guided, multidisciplinary interventions that could recalibrate KYN metabolic activity across cancer, mood, neurodegeneration, and metabolic disorders, appealing to clinicians, bioengineers, and systems biologists alike.

## 1. Introduction

**Figure 1:**
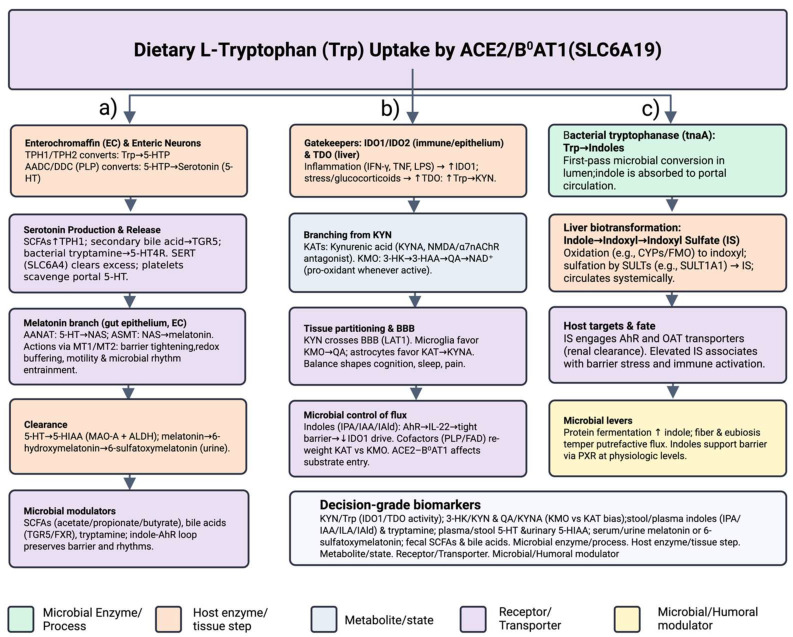
The gut microbiota–mediated partitioning of dietary tryptophan into serotonin–melatonin, kynurenine (KYN), and indole sulfate pathways. This schematic illustrates the major metabolic fates of dietary tryptophan (Trp) in the context of gut–microbiota–host interactions. Trp absorption via the ACE2/B^0^AT1 transporter complex sets the substrate pool for three principal routes: (**a**) the serotonin–melatonin pathway in enterochromaffin cells and enteric neurons, modulated by microbial metabolites such as short-chain fatty acids (SCFAs), bile acids, and tryptamine; (**b**) the KYN pathway initiated by indoleamine 2,3-dioxygenase (IDO) 1/IDO2 and tryptophan 2,3-dioxygenase (TDO), with downstream neuroactive branches toward kynurenic acid (KYNA) or quinolinic acid (QA), and regulated by immune tone, stress, and microbial indole–aryl hydrocarbon receptor (AhR) signaling; and (**c**) the microbial indole pathway, where bacterial tryptophanase produces indole, which is further metabolized in the liver to indoxyl sulfate (IS), a uremic toxin with AhR activity. Color coding distinguishes microbial steps, host enzymatic conversions, and intermediate metabolites, highlighting nodes where microbiota modulate flux and gut–brain signaling. AADC, aromatic L-amino acid decarboxylase; AANAT, arylalkylamine N-acetyltransferase; ACE2/B^0^AT1, angiotensin-converting enzyme 2/neutral amino acid transporter B^0^AT1; AhR, aryl hydrocarbon receptor; ALDH, aldehyde dehydrogenase; α7nAChR, alpha 7 nicotinic acetylcholine receptor; ASMT, acetylserotonin O-methyltransferase; BBB, blood–brain barrier; CYPs, cytochrome P450 enzymes; DDC, DOPA decarboxylase; EC, enterochromaffin cell; FAD, flavin adenine dinucleotide; FMO, flavin-containing monooxygenase; FXR, farnesoid X receptor; 3-HAA, 3-hydroxyanthranilic acid; 5-HIAA, 5-hydroxyindoleacetic acid; 3-HK, 3-hydroxykynurenine; 5-HTP, 5-hydroxytryptophan; 5-HT4R, 5-hydroxytryptamine Receptor 4; IAld, indole-3-aldehyde; IDO, indoleamine 2,3-dioxygenase; IL-22, interleukin-22; ILA, indole-3-lactic acid; INF-γ, interferon gamma; IPA, indole-3-propionic acid; IS, indoxyl sulfate; KATs, kynurenine aminotransferases; KMO, kynurenine 3-monooxygenase; KYN, kynurenine; KYNA, kynurenic acid; LAT1, L-type amino acid transporter 1; LPS, lipopolysaccharide; MAO-A, monoamine oxidase A; MT, melatonin receptor; NAD, nicotinamide adenine dinucleotide; NAS, N-acetylserotonin; NMDA, N-methyl-D-aspartate; OAT, organic anion transporter; PLP, pyridoxal-5′-phosphate; PXR, pregnane X receptor; SCFAs, short-chain fatty acids; SLC6A19, solute carrier family 6 member 19; SULTs, sulfotransferases; QA, quinolinic acid; SERT; serotonin transporter; SLC6A4, solute carrier family 6 member 4; TDO, tryptophan 2,3-dioxygenase; TGR5, Takeda G-protein–coupled receptor 5; TNF; tumor necrosis factor; TPH, tryptophan hydroxylase; Trp, tryptophan.

Life on Earth has been inextricably linked to a dynamic coexistence with microbes [1]. From the earliest stages of development through aging, humans are continuously shaped by microbial signals encountered through environmental contact, diet, and respiration [2]. Nowhere is this symbiosis more profound than in the gastrointestinal tract, where densely populated and metabolically diverse microbiota transform dietary substrates into bioactive molecules with far-reaching systemic effects—including on the brain [3]. Among the complex network of metabolic interactions bridging the gut and distant organs, the tryptophan (Trp) metabolic axis has emerged as a pivotal regulator of immune homeostasis, neurophysiological integrity, and energy balance [2,4]. Beyond serving as a precursor for serotonin and niacin, Trp is enzymatically channeled by both host and microbial systems into the kynurenine (KYN) metabolic pathway, generating a suite of metabolites capable of modulating inflammatory responses—either dampening immune activation or exacerbating pathological inflammation [5] (Figure 1). Additionally, natural compounds targeting neuroinflammation are gaining attention for their antidepressant potential, offering a complementary pathway to modulate Trp metabolism [6].

Over the past decade, next-generation sequencing and metabolomics have mapped thousands of associations between altered Trp metabolism and diseases as diverse as depression, diabetes, cancer, and Alzheimer’s disease (AD) [7,8]. Over the past decade, groundbreaking research has redefined our understanding of neurological diseases and mental illnesses, laying the groundwork for precision interventions [9,10]. Moreover, the integration of biomarkers and imaging with neuroinflammatory markers offers promising diagnostic and therapeutic insights in AD and related disorders [11]. Similarly, phytochemicals such as phenols, alkaloids, and terpenoids have demonstrated notable neuroprotective effects against neurodegenerative disorders, offering complementary intervention strategies [12,13,14,15]. Yet association is not causation [16]. We still lack a coherent framework linking specific microbial consortia, host enzymes such as indoleamine 2,3-dioxygenase 1 (IDO1) and tryptophan 2,3-dioxygenase (TDO), and downstream metabolites like kynurenic acid (KYNA) or quinolinic acid (QA) to discrete physiological outcomes [17] (Table 1). This gap hampers the design of targeted interventions—whether they involve probiotics, small-molecule inhibitors, or lifestyle prescriptions—that aim to rebalance Trp flux toward health-promoting routes [18]. Recent reconceptualizations propose a paradigm shift in how Trp–KYN metabolism is targeted for innovative clinical interventions [19].

The second challenge is spatial. Traditional bulk assays average signals across tissues and cell types, obscuring metabolic micro-domains that may act as “checkpoints” for Trp’s fate [20] (Table 1). Recent spatial omics and single-cell technologies have begun to reveal astrocytic and microglial niches in the human prefrontal cortex, endothelial “gates” at the blood–brain barrier (BBB), and perivascular hubs in peripheral organs where KYN metabolic activity is disproportionately high [21]. These findings demand a rewiring of our mental map: instead of a homogeneous pipeline, Trp metabolism resembles a switchboard with cell-type-specific levers that can be pharmacologically or genetically tuned [22].

Timing is the third frontier. Circadian biologists have long known that virtually every metabolic pathway oscillates over the 24-h day [22,23]. Emerging evidence suggests that KYN metabolism is no exception and that sex hormones further modulate these rhythms [24] (Table 1). Clinical trials of chemotherapy and checkpoint inhibitors demonstrate that dosing time can double efficacy or halve toxicity, yet KYN-targeting agents have rarely been tested under chronopharmacological designs [25]. Without granular, time-stamped metabolite monitoring, we risk missing critical windows when interventions would be most effective—or least harmful [26].

Technological advances now offer tools to close these knowledge gaps [27]. Stable-isotope tracing in gnotobiotic mice can timestamp flux through IDO1 versus TDO; single-cell proteomics in intestinal organoids can pinpoint which epithelial or immune subsets respond to specific “metabokines”; and clustered regularly interspaced short palindromic repeats (CRISPR)-based kill switches or inducible operons allow synthetic consortia to dial metabolite output like a volume knob [27,28,29]. Parallel progress in wearable biosensors, artificial intelligence (AI) feedback loops, and adaptive trial designs promises to link real-time biomarker readouts—such as saliva KYNA or morning KYN/Trp slopes—to dynamic adjustments in drug dose, exercise load, or probiotic composition [30,31,32].

Yet formidable obstacles remain. We lack validated, non-invasive biomarkers that faithfully mirror tissue-level KYN activity [33]. The ecological rules governing colonization by engineered microbes are not fully charted, and existing kill-switch circuits require rigorous containment testing [29]. Regulatory frameworks struggle to keep pace with live live biotherapeutic products (LBPs) that blur the line between drugs and devices [34]. Finally, ethical and logistical challenges complicate the deployment of adaptive, time-randomized clinical trials that integrate molecular, behavioral, and environmental data streams [35].

Against this backdrop, the present review aims to synthesize cutting-edge insights across microbiology, neuroscience, immunology, and bioengineering to articulate a unified roadmap for the precision modulation of the Trp–KYN axis. We first survey the spatial organization of KYN metabolism “checkpoints” in the brain and periphery, highlighting how localized enzyme activity interfaces with systemic immunity and neural circuitry. We then examine sex- and circadian-specific modifiers that dictate when and how the pathway tilts toward neurotoxicity or resilience. Next, we explore microbiota-based strategies—from designer consortia to encapsulated post-biotics—that act as precision switches for Trp flux, and we discuss their manufacturing, safety, and regulatory hurdles. Finally, we outline “Intervention 2.0”, an integrated platform combining dual-enzyme inhibitors, structured exercise, and AI-driven biosensing to create closed-loop therapeutics. By weaving these threads together, we seek to move the field beyond static snapshots toward dynamic, multi-scale models that can predict individual responses and guide adaptive interventions (Table 1). In doing so, we hope to catalyze collaborations between bench scientists, clinicians, data engineers, and regulatory experts, accelerating the translation of Trp-KYN biology into tangible health benefits.

The objectives of this review is to map the cellular and spatial heterogeneity of KYN metabolic activity and identify metabolic “checkpoints” amenable to intervention; to critically appraise evidence for the sex- and circadian-dependent modulation of Trp metabolism and its clinical implications; to evaluate current and emerging microbiota-based tools for the precision control of KYN flux, including synthetic consortia, kill switches, and post-biotic delivery systems; to assess the therapeutic promise and practical challenges of dual IDO1/TDO inhibition in conjunction with lifestyle levers such as exercise; and to propose a framework for adaptive, biomarker-guided clinical trials that integrate real-time metabolite monitoring with dosing algorithms. By addressing these aims, this review intends to illuminate fertile research avenues and provide a practical blueprint for next-generation strategies targeting the gut–brain–immune axis via the KYN metabolic pathway.

## 2. Microbiota-Driven Modulation of Indoleamine 2,3-Dioxygenase 1 (IDO1) and Tryptophan 2,3-Dioxygenase (TDO) Signaling

**Figure 2:**
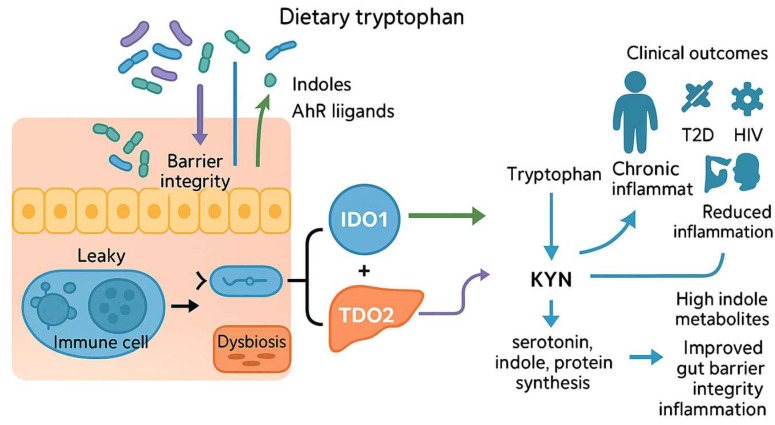
The microbial “remote control” of indoleamine 2,3-dioxygenase 1 (IDO1) and tryptophan 2,3-dioxygenase (TDO) enzyme signaling. Visual representation of how gut-derived microbial metabolites (indoles, SCFAs, and AhR ligands) modulate the enzymatic activity of IDO1 and TDO. This figure emphasizes the bidirectional interplay between intestinal microbiota, barrier integrity, immune signaling, and systemic health outcomes. AhR, aryl hydrocarbon receptor; HIV, human immunodeficiency virus; IDO1, indoleamine 2,3-dioxygenase 1; KYN, kynurenine; SCFAs, short-chain fatty acids; TDO, tryptophan 2,3-dioxygenase; T2D, type 2 diabetes.

Our intestinal microbiota function like molecular “remote controls,” fine-tuning dietary Trp’s fate before it enters systemic circulation [4,36] (Figure 2). Through sophisticated fermentation and metabolic processing, gut bacteria generate bioactive molecules such as indole derivatives, short-chain fatty acids (SCFAs), and aryl hydrocarbon receptor (AhR) ligands [36,37]. These small signaling molecules migrate to immune and hepatic tissues, influencing critical enzymes like IDO1 and TDO [38]. Consequently, Trp metabolism shifts: toward the KYN pathway when these enzymes are active, or towards serotonin, indole, and protein synthesis when their activity is inhibited [39]. Clinical and experimental studies have connected microbiota-driven dysbiosis and altered metabolite profiles to chronic inflammation and diseases including obesity, type 2 diabetes, human immunodeficiency virus (HIV), colorectal cancer, and AD [40,41,42,43,44]. An elevated KYN/Trp ratio, indicative of heightened IDO1 activity, is associated with compromised gut barrier integrity and systemic immune activation [45]. Conversely, higher levels of indole metabolites correlate with strengthened epithelial barriers and reduced inflammation [46]. Dietary interventions, particularly promoting fiber-rich taxa such as Bifidobacterium and Lactobacillus, enhance protective indole and short-chain fatty acid production, counteracting excessive KYN synthesis and promoting healthier metabolic pathways [47].

### 2.1. Literature Review: Microbial Metabolites as Modulators of Intestinal Integrity and Systemic Disease

Gut microbiota profoundly influence host health through their role in Trp metabolism [48]. Microbial metabolites like KYN, tryptamine, and indole-3-propionic acid regulate critical pathways related to immune, neurological, and metabolic health [49]. Notably, microbiota-produced KYN derivatives significantly impact intestinal permeability and obesity, highlighting their broad physiological relevance [50]. Additionally, these microbial metabolites demonstrate both pro- and anti-inflammatory effects, positioning them as promising therapeutic agents for managing intestinal inflammation and associated disorders, including inflammatory bowel disease and irritable bowel syndrome [51]. Dysbiosis-induced disruptions in Trp metabolism have also been implicated in autoimmune disorders and ischemic stroke, suggesting significant clinical implications and potential therapeutic benefits of dietary strategies involving food homologous plants [17,52]. Germ-free animal models further underline the essential role of microbiota in guiding host Trp metabolism, reinforcing the microbiome’s critical function as a regulator of health, aging processes, and disease progression [53].

### 2.2. Research Gaps: Gaps in Dosing Strategies, Longitudinal Efficacy, and Mechanistic Insights

Current research gaps include the need for clearer insights into optimal dosing and intervention strategies, particularly in clinical applications such as haploidentical transplantation, where long-term outcome data remain limited [54]. Additionally, the precise mechanisms underlying sustained benefits, as demonstrated in long-term diabetes remission trials, require further exploration to enhance replicability and scalability [55]. In educational and psychological contexts, while significant improvements in creativity through musical interventions are documented, there is still a limited understanding of long-term impacts and the comparative effectiveness of different teaching methodologies [56]. Moreover, longitudinal health outcome studies, such as those examining coronavirus disease 2019 (COVID-19) survivors, underscore the necessity of extended follow-ups to better understand persistent symptoms and recovery trajectories, emphasizing gaps in comprehending chronic implications [57]. Taken together, these findings underscore the need for extended longitudinal, comparative, and mechanistically oriented studies across clinical, educational, and therapeutic domains to refine and empirically validate intervention efficacy [58].

These gaps in dosing and efficacy cannot be disentangled from the complex regulatory role of gut microbiota in shaping KYN metabolism. Microbial communities influence both the magnitude and duration of IDO1/TDO activation through the dynamic production of signaling molecules like SCFAs, AhR ligands, and indoles [47,59,60,61]. These microbial signals fluctuate with diet, circadian rhythm, antibiotic exposure, and host immune tone—factors that vary over time and across individuals [62]. Consequently, a static, one-size-fits-all intervention may fail to accommodate these microbial dynamics, explaining the inconsistent long-term outcomes observed in clinical trials [63]. Moreover, the lack of mechanistic clarity—especially regarding which microbial species modulate which enzymatic checkpoints—limits our ability to rationally time or personalize interventions [62,64]. Without integrating gut microbiota profiles and temporal shifts in microbial metabolite output, dosing strategies remain empirical and imprecise [65]. Bridging this mechanistic blind spot is essential if we hope to evolve microbiota-modulating interventions from broad-spectrum dietary tweaks into finely tuned therapeutic levers within the KYN pathway [65,66,67].

### 2.3. Time-Stamped Isotope Tracing in Gnotobiotic Mice Can Tag Flux Through Indoleamine 2,3-Dioxygenase 1 (IDO1) Versus Tryptophan 2,3-Dioxygenase (TDO)

Emerging research highlights crucial insights into precisely distinguishing Trp metabolism fluxes mediated by IDO1 and TDO, especially within the context of cancer progression and immune modulation [68,69]. Advanced techniques such as time-stamped isotope tracing in gnotobiotic mice offer significant promise for accurately mapping metabolic activity between these pathways [68,70,71]. Current evidence indicates that the dual inhibition of IDO1 and TDO surpasses single-target strategies, effectively reducing immunosuppressive KYN metabolites and enhancing antitumor immune responses across various cancers [72,73]. Nonetheless, substantial gaps remain in understanding precise metabolic dynamics, particularly in humanized mouse models designed to mimic human microbiota interactions [73,74]. Further insights have been gleaned from enzyme knockout studies demonstrating how the disruption of KYN pathway genes impacts mitochondrial respiration and energy homeostasis in the brain [75]. Recent knockout models targeting kynurenine aminotransferase (KAT) enzymes have revealed heightened oxidative and excitatory stress responses, offering mechanistic insights into depression and post-traumatic stress disorder-like behaviors [76]. Meanwhile, rational drug design targeting the KYN pathway is being explored to fine-tune neuroprotective outcomes without tipping the balance toward neurotoxicity [77]. Future research should prioritize detailed mechanistic exploration through robust isotope tracing methodologies, enabling a clearer delineation of the specific roles that IDO1 and TDO play in disease pathology [72,78]. Additionally, the further development and clinical validation of potent dual IDO1/TDO inhibitors are the critical next steps to fully harness their therapeutic potential and optimize immunotherapeutic strategies across multiple malignancies [79].

### 2.4. Single-Cell Proteomics in Intestinal Organoids Could Reveal Which Epithelial or Immune Subsets Sense Each “Metabokine”

Emerging research underscores the potential of single-cell proteomics in intestinal organoids as a powerful tool for clarifying which epithelial or immune subsets respond to specific “metabokines” [80,81,82]. Single-cell resolution can unveil distinct proteomic signatures, enabling the precise identification of cells sensing these metabolic signaling molecules [80,83]. Recent advancements demonstrate that intestinal organoids effectively model complex physiological responses, such as ischemia–reperfusion injury and gluten-induced inflammation seen in celiac disease, highlighting their suitability for detailed proteomic investigations [84,85,86]. Additionally, single-cell transcriptomics has successfully characterized specialized enteroendocrine cell populations, further emphasizing the feasibility and importance of single-cell analyses in organoid cultures [87,88,89,90]. Nevertheless, substantial research gaps persist in connecting specific metabolites or “metabokines” to their target cell types [80,81,82,91]. Future studies should employ single-cell proteomics to systematically map interactions between these bioactive metabolites and distinct cellular subsets within organoid models [80,92,93,94]. The crucial next steps involve refining protocols for isolating and analyzing pure populations of epithelial and immune cells, as well as developing robust analytical methods to integrate proteomic and transcriptomic data [87,95,96,97]. This integrated approach promises deeper insights into gut physiology and pathology, guiding targeted therapeutic interventions for intestinal and metabolic disorders [80,81,98,99].

### 2.5. Synthetic Consortia with Inducible Kynurenine (KYN) Operons Would Let Us Dial Metabolite Output Like a Volume Knob

Emerging research highlights the potential of synthetic microbial consortia with inducible KYN operons as sophisticated tools for precisely tuning metabolite production [100]. Utilizing inducible gene expression systems, these engineered microbial communities allow metabolite output to be modulated like a “volume knob,” providing highly controlled, dynamic responses tailored to specific applications [101]. Studies employing synthetic consortia, particularly those leveraging cross-feeding and inducible metabolic pathways, demonstrate their capacity for the robust and tunable synthesis of desired compounds, such as antioxidants and secondary metabolites [102]. For instance, yeast-based consortia effectively enhanced resveratrol production by precisely managing metabolic interactions. Further, advancements in understanding inducer–producer systems and integrating genomic, proteomic, and machine learning approaches have improved consortium stability, efficiency, and predictability [103,104]. Despite promising initial results, substantial work needs to be conducted in optimizing consortia dynamics, including refining inducer responsiveness, ensuring population stability, and developing standardized, scalable frameworks [105]. The next steps should focus on advanced computational modeling and experimental validation, employing genome-wide screens and emerging technologies to fine-tune induction mechanisms [106]. Ultimately, synthetic consortia with precisely controlled KYN operons hold transformative potential for targeted metabolic engineering and biomedical applications.

### 2.6. Molecular Mechanisms Linking Gut Microbiota to Kynurenine (KYN) Pathway Enzymes

The gut microbiota orchestrates KYN metabolic activity through a network of direct molecular interactions that go beyond metabolite secretion [107,108,109]. Certain microbial strains modulate host the expression of IDO1 and TDO via Toll-like receptor (TLR) engagement, type I interferon signaling, and NF-κB activation, tipping Trp metabolism toward immunosuppressive KYN derivatives [107,110,111]. For instance, *Bacteroides fragilis* activates TLR2, which upregulates IDO1 in dendritic cells, while *Lactobacillus reuteri* can suppress IDO1 through IL-10-mediated signal transducer and activator of transcription 3 (STAT3) signaling [108,109,112]. AhR ligands such as indole-3-aldehyde not only shape T-cell differentiation but also transcriptionally repress TDO via microRNA-132/212 axes in hepatocytes [109,113,114]. Bacterial-derived SCFAs (e.g., butyrate) inhibit histone deacetylases, opening IDO1 promoter regions to cytokine-inducible enhancers [111,115,116]. Moreover, cross-feeding consortia modulate nicotinamide adenine dinucleotide (NAD) biosynthesis, indirectly affecting KYN flux through feedback loops involving SIRT1 and PGC-1α [109,112,117]. These mechanisms illustrate that microbiota–KP interactions are not merely passive but involve a bidirectional, cell-type-specific signaling web that governs systemic immune tone and neuroinflammation risk.

## 3. Kynurenine (KYN) Metabolic Pathway “Checkpoints” in the Brain’s Cellular Grid

**Figure 3:**
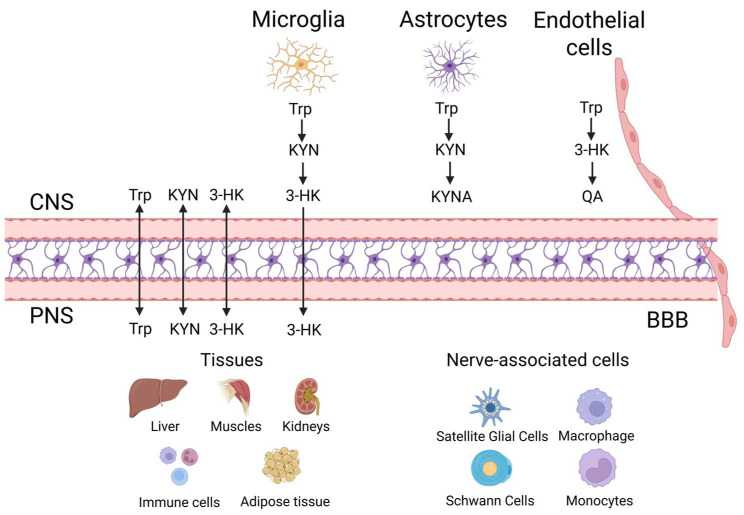
Spatial mapping of kynurenine (KYN) metabolism across the central nervous system (CNS) and peripheral nervous system (PNS). An illustration showing cellular-level checkpoints of KYN metabolism within the neurovascular unit. Distinct metabolic niches in microglia, astrocytes, and endothelial cells are depicted, highlighting their roles in regulating immune surveillance, metabolic flux, and neuronal resilience, particularly at the blood–brain barrier (BBB). BBB, blood–brain barrier; 3-HK, 3-hydroxykynurenine; CNS, central nervous system; KYN, kynurenine; KYNA, kynurenic acid; PNS, peripheral nervous system; QA, quinolinic acid; Trp, tryptophan.

The brain’s intricate cellular network is governed by distinct “checkpoints” that regulate critical metabolic pathways, notably the KYN metabolic pathway [118] (Figure 3). Recent advancements using spatially resolved omics have uncovered specialized microglial and astrocytic niches within the human prefrontal cortex, which display notably elevated activity toward KYN metabolism [119]. Additionally, specific endothelial clusters located at neurovascular units show significant KYN metabolic signatures, suggesting their crucial roles as regulatory gates controlling permeability and transport functions at the BBB [120]. These novel insights provide a detailed spatial mapping of KYN metabolism modulation across the brain’s cellular landscape, emphasizing its essential function in neuroimmune communication and neuronal homeostasis [121]. Advances in Parkinson’s disease research underscore how metabolic dysregulation, particularly in mitochondrial and KYN pathways, contributes to neurodegeneration [122]. Elucidating the precise locations and mechanisms of these KYN checkpoints enhances our understanding of how Trp metabolism contributes to maintaining brain health and how disruptions in this pathway might underpin various neurological and psychiatric disorders [123]. Disruptions in KYN metabolism have also been implicated in age-associated vascular dysfunction and sarcopenia, further linking metabolic decline to cognitive vulnerability [124]. Evidence from metabolic interventions, including AdipoRon treatment, supports the notion that correcting mitochondrial and inflammatory dysfunctions can mitigate neurodegenerative processes [125]. Future research focusing on these checkpoint regions promises innovative approaches to therapeutic interventions aimed at adjusting KYN activity, offering potential improvements in managing neurological health and disease [126,127,128,129].

### 3.1. Literature Review: Mapping Kynurenine (KYN) Dynamics Across Neurovascular and Immune Landscapes

KYN “checkpoints” distribute across the brain and periphery, steering Trp catabolism and dictating immune tone [130]. Spatial omics now map microglial, astrocytic, and endothelial niches in the human prefrontal cortex where KYN enzymes are up-shifted, flagging BBB gates that couple metabolism with neuroimmune signaling [131]. At these hubs, surges in KYN dampen neuronal resilience, promote cerebrovascular inflammation, and correlate with depression, dementia, and stroke [132]. Outside the CNS, the same metabolic nodes calibrate T-cell fitness [133]. IDO1 overactivation floods tumors with immunosuppressive KYN, paralyzing cytotoxic responses; yet IDO1 inhibitors alone have disappointed, unveiling redundant escape circuits [134]. Environmental pollutants and viral infections further hack these checkpoints, amplifying cytokine storms by tipping regulatory T-cell (Treg)/T helper 17 cell (Th17) balance [135]. Conversely, systemic checkpoints safeguard Trp homeostasis; deficiency or gene defects can trigger hypertryptophanemia, disturbing serotonin and niacin biosynthesis [133]. Next-wave interventions pair metabolic brakes with programmed cell death protein 1 (PD-1)/programmed cell death ligand 1 (PD-L1) or heat shock protein 90 (HSP90) blockade, deploy AhR antagonists, phytochemicals, or AhR inhibitors such as BAY2416964 to restore immunity and curb tumor growth [136]. These innovative models, complemented by optogenetic technologies, represent the forefront of translational neuroscience [137]. Brain-penetrant molecules that selectively damp microglial KYN flux while sparing peripheral metabolism could deliver precision therapy for neuropsychiatric disorders [138]. Integrating multi-omics with machine learning will rank checkpoint hierarchies, predict compensatory loops, and guide patient-specific cocktails that re-balance neuroimmune networks and prevent KYN-driven pathology [139].

### 3.2. Research Gaps: Mapping, Monitoring, and Modulating Kynurenine (KYN) Checkpoints Across Systems

KYN checkpoints—specialized microglial, astrocytic, and endothelial clusters—regulate Trp catabolism and immune tone across the brain–body axis [140]. Spatial omics pinpoints these hubs in the human prefrontal cortex and BBB, where KYN overload erodes neuronal resilience and fuels depression, stroke, and dementia [141]. Peripheral checkpoints likewise calibrate T-cell vigor [140,142]. Tumors hijack the circuit: chronic IDO1 activity floods the microenvironment with immunosuppressive KYN, yet IDO1 blockade alone is insufficient as it can be bypassed by TDO and alternative AhR ligands [134,143]. Environmental toxins, infections, and diet further affect these metabolic gates, whereas systemic checkpoints defend serotonin and niacin pools [144]. Critical gaps stall translation. Cell-type-resolved maps of IDO1, TDO, transporters, and System L remain incomplete; single-cell, longitudinal multi-omics in inflamed tissues and cancers are needed [141,145]. Real-time biomarkers of KYN burden and transporter flux are lacking, complicating precision dosing [141,146]. Optimal regimens for dual IDO1/TDO or AhR blockade—and for brain-penetrant microglial modulators—are undefined [147]. Preclinical models rarely integrate microbiota composition, circadian rhythm, sex, or pollutant exposure, all strong modifiers of KYN balance [74,144,145]. Bridging these voids will require integrated biosensing, adaptive trials, and machine learning-guided strategies to tailor checkpoint-directed therapies to individual neuroimmune landscapes [141,148,149,150].

### 3.3. Clustered Regularly Interspaced Short Palindromic Repeats Interference (CRISPRi) “Zip-Codes” Delivered by Adeno-Associated Virus (AAV) Can Silence Kynurenine 3-Monooxygenase (KMO) or Kynureninase (KYNU) Only in Perivascular Endothelium and Watch Downstream Glutamatergic Sync Crash—or Not

Picture an adeno-associated virus (AAV) vector outfitted with endothelial “zip-codes”—tight-junction promoters plus microRNA target sequences for off-cell clearance—delivering a zinc finger protein 3 (ZIM3)-Kruppel-associated box (KRAB) clustered regularly interspaced short palindromic repeats interference (CRISPRi) cassette that knocks down kynurenine 3-monooxygenase (KMO) or kynureninase (KYNU) exclusively in perivascular endothelium [151,152,153,154]. Two factors drive this strategy. First, perivascular cells are gatekeepers for KYN flux into the brain: endothelium converts circulating KYN into 3-HK and QA, feeding astrocyte–microglia glutamatergic coupling [155]. Second, KMO overactivity fuels cancer growth, neurodegeneration, and immune escape, yet systemic inhibition risks off-target NAD imbalance [156]. Cell-restricted CRISPRi sidesteps that liability, and the ZIM3 domain offers potent, reversible silencing [157]. Sex- and circadian-modulated shifts in neurodegeneration also modulate vulnerability to cognitive and mood disturbances [158]. If endothelial KMO/KYNU expression drops, 3-HK and QA output should collapse, starving downstream N-methyl-D-aspartate (NMDA)-sensitizing signals and “crashing” the synchronized glutamatergic surge that drives excitotoxicity and tumor immune evasion [159]. The next steps include building bar-coded AAV libraries to refine endothelial specificity; validating knock-down efficiency and KYN metabolites flux in brain slice co-culture; monitoring glutamate dynamics with optogenetic reporters in vivo; and assessing effects on tumor infiltration and behavior in KMO-high breast-cancer metastasis models [155,160,161]. Parallel safety screens must chart NAD pools and mitochondrial stress in non-target tissues [162] (Table 2). Successfully doing so could provide the precise means to modulate neurovascular KYN flux without systemic collateral damage [151,155,160].

### 3.4. Light-Addressable Riboswitches Could Let Us Pulse Kynurenine (KYN) Enzymes in Astrocytes and Read Real-Time Calcium Waves

Light-addressable riboswitches offer a precision dimmer for the KYN metabolic pathway inside astrocytes [163]. By coupling a photocleavable aptamer to KMO or KYNU transcripts, pulsed infrared or nanosecond-visible light can switch enzyme translation on or off within milliseconds, riding on the same wavelengths already proven to trigger or image astrocytic Ca^2+^ waves [164]. The concept rests on two converging clues: riboswitches endowed with the Z-lock or photocleavable linker reliably gate gene expression in living cells, and pulsed light drives robust, tunable calcium oscillations in astrocytes without overt photodamage, letting us read the metabolic consequences in real time [165] (Table 2). A burst of light thus delivers a double payload—inducing a KYN metabolism surge while simultaneously recording its impact through a genetically encoded calcium indicator based on calmodulin and M13 peptide fused to green fluorescent protein (GCaMP)-tagged calcium reporters or two-photon glutamate sensors [165,166]. The key next steps include engineering astrocyte-specific AAVs carrying the light-gated riboswitch, benchmarking translation kinetics versus calcium rise in organotypic slices, and mapping how localized KYN pulses propagate through extracellular gliotransmitter waves [166,167]. In vivo, fiber-coupled two-photon uncaging combined with wide-field Ca^2+^ imaging can reveal whether transient KMO activation amplifies or quenches network excitability during sleep, seizure, or learning [165,168]. Ultimately, this optogenetic–metabolic fusion could dissect causality between KYN flux and astrocyte-driven neurophysiology with unprecedented temporal resolution [163,169,170].

## 4. Sex and the Circadian City: Hidden Modifiers

**Figure 4:**
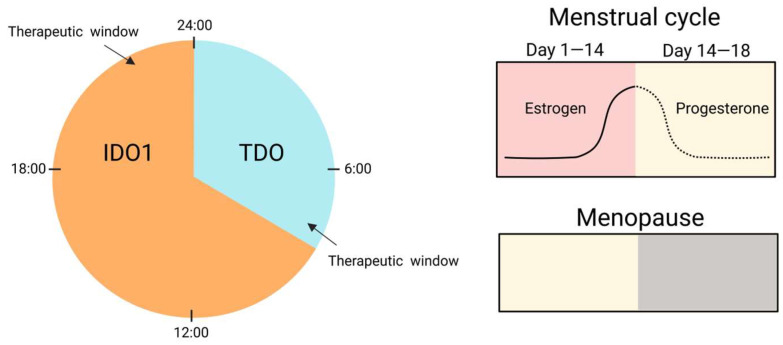
The chronotherapeutic and sex-specific modulation of the kynurenine (KYN) metabolic pathway. A circadian wheel and hormonal timeline showing sex-specific differences in KYN metabolism, immune sensitivity, and therapeutic windows. This figure captures how time of day and hormonal phases (the menstrual cycle or menopause) can alter the efficacy and safety of interventions targeting KYN pathway enzymes.

Circadian rhythms and biological sex provide an under-appreciated backdrop to KYN metabolism, subtly steering mood and immune tone across the day [171]. Sex hormones and circadian rhythms likewise modulate migraine vulnerability, further emphasizing the need for personalized neuroimmune interventions [172]. Immune challenges such as interferon-α therapy consistently lower circulating Trp, boost KYN, and heighten depressive symptoms, but these shifts peak at distinct clock phases and manifest more severely in women [173]. Preclinical lipopolysaccharide models echo this dimorphism, with female mice displaying protracted neuroimmune and behavioral scars that fluoxetine can erase only when given at their active phase [174]. Human imaging studies add another layer: reduced Trp and skewed KYN metabolites are associated with hippocampal-subfield atrophy, yet the correlation strengthens in early morning scans, hinting at chronobiological gating [175]. Rapid-acting antidepressants such as ketamine appear to reset both the monoaminergic–glutamatergic interface and downstream KYN flux, but again, response rates diverge by sex and time of dosing [173,176]. Post-mortem data reveal anterior cingulate cortex KYN signatures that segregate by sex and suicide status, underscoring personalized vulnerability windows [175,177]. Neuroanatomical findings from autism research similarly reveal that circadian and neuroimmune factors may influence structural brain development [178] (Figure 4). Collectively, these findings suggest that sex hormones and the molecular clock act as “hidden modifiers,” dictating when and how immune activation tilts KYN balance toward neurotoxicity or resilience [179]. Similarly to the evolving understanding in mental health research, recognizing sex- and circadian-dependent variability supports a dimensional view of disease vulnerability and resilience [180]. Parsing these temporal–sexual intersections could unlock chronotherapy strategies for mood disorders.

### 4.1. Literature Review: Circadian Misalignment (CM), Mood Vulnerability, and Emerging Chronotherapeutics

Longitudinal wearables reveal that phase delays in core-body temperature and melatonin release often precede mood dips in major depression and bipolar disorder, supporting circadian realignment as a digital medicine target [181]. In healthy adults, endogenous 24 h rhythms modulate anxiety- and depression-like effects, with later dim-light melatonin onset and compressed phase-angle differences predicting poorer mood [182]. The disruption of the molecular clock by hypercortisolism in Cushing’s syndrome flattens immune cell oscillations and skews steroid profiles, illustrating endocrine–immune crosstalk [183]. Similar circadian misalignment (CM) exacerbates autoimmunity in lupus, acting as a prodromal biomarker for flares [184]. Non-pharmacologic interventions show promise: group music therapy realigns autonomic and melatonin rhythms in depressed women, while optimal circadian–circasemidian coupling buffers morning blood pressure surges and fosters resilience [182,185]. Nutritional modulation is equivocal; omega-3 supplementation dampens inflammation yet leaves KYN metabolism and mood unchanged in healthy men [186]. Experimental LPS infusion acutely activates KYN metabolism, linking immune challenge, chronobiology, and effect in real time [186]. Adolescents display bidirectional pathways: anxiety forecasts future sleep disruption, whereas a genetically longer free-running circadian period in males predicts later mood vulnerability [184,185]. Collectively, these studies underscore circadian alignment as a modifiable lever for mood regulation and highlight the need for personalized chronotherapeutic strategies across lifespan and disease contexts.

### 4.2. Research Gaps: Timing, Sex, and Biomarker Integration for Precision Kynurenine (KYN) Intervention

Despite compelling evidence that dosing time and sex strongly influence chemotherapy and immunotherapy outcomes, major knowledge gaps limit translation to KYN-targeting agents [187]. First, no clinical trials have yet tested chronopharmacology for IDO1/TDO inhibitors, KMO blockers, or KAT activators, leaving optimal schedules unexplored [188]. Second, existing studies rarely stratify by both circadian phase and biological sex; pharmacokinetic data disaggregated for women are virtually absent, obscuring why afternoon regimens reduce toxicity in female lymphoma patients [187]. Third, mechanistic links between peripheral clock genes, immune cell oscillations, and drug metabolism remain undefined, particularly how estrogen and glucocorticoids modulate daily fluctuations in KYN enzyme activity [189]. Fourth, wearable-derived chronotypes are not integrated into trial design, preventing personalized timing algorithms that could harmonize drug exposure with individual rhythms [187]. Fifth, preclinical tumor models often use male rodents housed in static light cycles, overlooking sex- and circadian-dimorphic responses seen in patients [190]. Finally, validated real-time biomarkers that couple KYN metabolite swings to treatment efficacy are lacking, hindering adaptive dosing [191] (Table 3). Addressing these gaps will require multi-omics chronopharmacology studies, sex-balanced animal models, and adaptive clinical trials embedding circadian sensors to craft precision schedules for KYN-focused therapies.

### 4.3. Multi-Time-Point Plasma Kynurenine (KYN) Profiles Stratified by Sex and Hormonal Phase

Current evidence shows that plasma KYN levels fluctuate with sex, age, and endocrine status, yet most datasets rely on single fasting draws [192]. Parkinson’s and breast cancer cohorts reveal disease-specific shifts toward neurotoxic QA, but without high-resolution sampling, it is unclear whether these changes reflect trait abnormalities or time-of-day/hormone-phase effects [193]. Metabolomic screens (KarMeN) can classify sex and age, implying a strong biological signal, while ambulatory microdialysis (U-RHYTHM) now permits hourly hormone/metabolite capture—an underused tool for KYN kinetics [193]. Feminizing gender-affirming hormone therapy reshapes amino acid pools after six months; however, acute diurnal patterns and menstrual cycle nuances remain unmapped [194]. Animal work shows estrous-dependent adipokine oscillations, hinting that luteal-phase progesterone surges might also bias KYN flux [194]. No study yet aligns luteal sub-phases, cytokine pulses, and KYN derivatives in healthy women, nor measures phase-specific KYN shifts during immunotherapy or antiandrogen treatment [192]. The next steps: deploy wearable-triggered, multi-time-point plasma collection across 24 h and menstrual cycles; integrate liquid chromatography–mass spectrometry (LC-MS) panels for KYN, Trp, QA, and KYNA with sex hormone, cortisol, and brain-derived neurotrophic factor (BDNF) profiles; and model trajectories using mixed-effects chronopharmacology frameworks. Such datasets will clarify whether timing and hormonal milieu confound or mediate KYN biomarkers, guiding precise sampling windows and sex hormone-aware interventions.

### 4.4. Wearable Light Exposure + Metabolite Logging to See If Circadian Misalignment (CM) Exaggerates the Quinolinic Acid (QA) Spike

Circadian misalignment (CM) rewires metabolic, cardiovascular, and immune circuits, yet whether it magnifies neurotoxic (QA) surges remains untested [195]. Night-shift paradigms reveal clock-driven insulin resistance, lipidome disruption, and blood pressure creep, while murine models link chronic CM to immune senescence and shortened lifespan [196]. Sex adds complexity—females show partial protection against CM on a high-fat diet—suggesting divergent QA trajectories [197]. Light timing is the master zeitgeber; continuous lux logging via smartwatches can quantify misalignment magnitude, whereas ambulatory microdialysis or dried-blood-spot kits now enable multi-time-point KYN metabolite sampling [184,198]. Pairing these technologies would let researchers correlate light-phase offsets with 24 h QA area-under-the-curve values, stratified by sex and feeding rhythms [199]. The key next steps include piloting a cross-over study where shift workers wear light and activity trackers plus collecting hourly capillary samples across two work cycles; modeling QA dynamics versus lux-derived phase angles using mixed-effects chronobiology; overlaying cortisol and melatonin rhythms to disentangle stress versus circadian effects; and testing whether timed blue-light blockers, melatonin, or time-restricted feeding blunt QA spikes [199,200] (Table 3). Such integrative phenotyping could identify high-risk chronotypes and guide precision-timed KYN interventions to mitigate CM-induced neuroinflammation.

### 4.5. Adaptive Trial Designs That Randomize Dose Timing Rather than Just Dose Size

Bayesian and response-adaptive frameworks have revolutionized dose finding, yet virtually all published trials modulate quantity, not clock time [201]. Radiation for pancreatic cancer, ketamine infusions for late-life depression, and molnupiravir for early COVID-19 show how real-time efficacy–toxicity trade-offs hone dose size, but none test whether morning versus evening delivery alters these curves [202]. Chronotherapy evidence from chemotherapy and immune checkpoint inhibitors indicates that timing can double efficacy or halve toxicity, with sex as a major moderator, underscoring a missed opportunity [203]. Key gaps include statistical models that treat dosing time as a continuous, circadian-anchored covariate; real-time biomarkers (e.g., actigraphy-derived phase angle) to guide allocation; and operational logistics for pharmacy and nursing around-the-clock interventions [201,204]. The next steps include simulating Bayesian hierarchical designs where dose level and dosing hour are co-randomized, borrowing strength across adjacent time bins; integrating wearable-captured chronotypes to stratify randomization and inform priors; embedding rolling interim analyses that reduce unfavorable time windows rather than doses; and piloting such designs in drugs with known chronotoxicities, using point-of-care melatonin or cortisol assays as safety triggers [202,205] (Table 4). Developing regulatory guidance and EHR-linked scheduling tools will be crucial to mainstream adaptive chrono-trials, paving the way for precision-timed KYN inhibitors and beyond [203,206].

## 5. Microbiota Engineering as a Precision Switch

**Figure 5:**
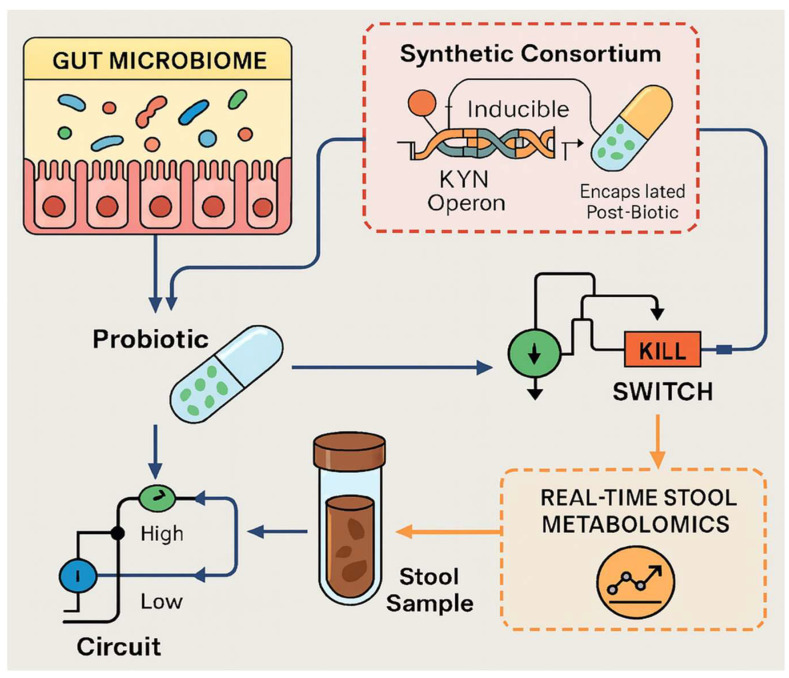
Precision modulation via engineered and smart intervention. Conceptual visualization of microbiota engineering strategies for precision intervention. Highlights include synthetic consortia with inducible kynurenine (KYN) operons, encapsulated post-biotics (e.g., stabilized kynurenic acid), kill switch technologies, and adaptive probiotic titration guided by real-time stool metabolomics.

Engineering the gut microbiome now offers a “precision switch” for neuroimmune circuits, with strain-level interventions poised to toggle mood and metabolic health on demand [207]. In murine models of chronic stress, supplementation with *Bifidobacterium pseudonumeratum* W112 reversed anhedonia and hepatic injury by recalibrating the gut–liver–brain axis, whereas *B. breve* and *B. longum* strains restored brain-derived neurotrophic factor signaling and serotonergic precursors through 5-hydroxy-tryptophan synthesis [208] (Figure 5). These psychobiotic effects align with peripheral data: patients with major depressive disorder show depleted *Bifidobacterium* and *Lactobacillus* counts, implicating taxa scarcity as a modifiable risk [209]. Importantly, antidepressive benefits persist even with heat-killed cells, underscoring that microbial metabolites—not colonization per se—drive behavioral rescue [208]. Precision editing could therefore install synthetic operons for tryptophanase, KATs, or short-chain fatty acid production to steer host Trp flux toward neuroprotective pathways and away from QA toxicity [210]. The key next steps include using high-resolution metagenomics to map strain-specific metabolic fingerprints, CRISPR-guided promoter tuning for inducible output, and gnotobiotic “plug-and-play” consortia to test combinatorial logic [211]. The adjunctive use of compounds like curcumin has been associated with improvements in microbiota composition and mood, reinforcing its role in gut–brain axis modulation [212]. Longitudinal trials integrating fecal metabolomics with mood and liver biomarkers will validate whether engineered consortia can act as dosage-controlled switches, heralding tailor-made microbiota therapeutics for depression and comorbid metabolic disease [213].

### 5.1. Literature Review: Microbiota-Targeted Strategies for Modulating Mood and Inflammation

Clinical and preclinical data increasingly connect gut-directed interventions to mood improvement [214,215]. A randomized trial showed *Bifidobacterium breve* CCFM1025 alleviates major depression while normalizing Trp-KYN balance and microbial diversity [216,217]. Complementary protocols—PRO-DEMET and PROVIT—deploy *B. longum*, *L. helveticus*, and biotin to probe combined metabolic–microbiome effects; preliminary findings suggest inflammation drops even when mood shifts are modest [216,218]. Beyond bifidobacteria, *Akkermansia muciniphila* and *Lactobacillus reuteri* mitigate stress-induced anhedonia in mice, accompanied by restored neurotrophins and lipid metabolism [216,219,220]. A single FMT case demonstrates symptom remission in refractory depression, echoing murine transfers where microbiota from depressed women induce affective and fatty acid perturbations in recipients [215,216,220]. Non-bacterial strategies also show promise: low-dose linoleic acid rescues serotonin and microbial diversity, while Banxia Xiexin decoction adjusts the gut–lipid axis to relieve comorbid atherosclerosis and depression [216,220,221]. Psychosocial immersion programs reveal that Prevotella-rich shifts correlate with lowered inflammatory tone and mood gains, underscoring environment–microbiome interplay [217,218,221]. Collectively, these studies depict a converging theme: targeted modulation—whether via single strains, multi-strain consortia, diet, or behavioral change—can rebalance microbial metabolites, dampen inflammation, and lift mood. Future trials must standardize endpoints, stratify by metabolic comorbidity, and incorporate multi-omics to pinpoint responders and refine dosing regimens.

### 5.2. Research Gaps: Live Biotherapeutic Products (LBPs) Against Multi-Drug Resistant Enteric Pathogens: Research Gaps

Proof-of-concept studies show engineered or defined consortia can decolonize carbapenem-resistant *Klebsiella* and other multi-drug resestant organisms without disrupting resident flora, yet translation stalls on several fronts [222]. First, colonization remains unpredictable: VE303 engrafts only after antibiotic conditioning, while VE707′s murine efficacy lacks human pharmacokinetic analogs [223]. No head-to-head trials compare synthetic consortia with FMT, leaving efficacy drivers—bacteriocins, phages, or niche competition—unclear [224]. Second, durability data are scarce; longitudinal sequencing in patients post-FMT hints at phage-mediated suppression, but the mechanistic dissection of phage–bacteria–host interplay is missing [225]. Third, safety and horizontal-gene-transfer risks are under-characterized—engineered *E. coli* secreting microcins could acquire resistance cassettes in vivo [226]. Fourth, manufacturing and QC frameworks lag behind pharmaceutical standards; batch-to-batch metabolite output is unverified for multi-strain LBPs [223]. Fifth, adaptive trial designs that modulate dose timing relative to antibiotics or diet are untapped, despite evidence that feeding rhythms and bile acids gate colonization resistance [227]. Finally, regulatory pathways for genetically modified LBPs remain fragmented across jurisdictions, deterring investment [223,228] (Table 5). Addressing these gaps demands standardized engraftment biomarkers, phage omics integration, the gnotobiotic validation of mechanism, and harmonized GMP guidelines to accelerate safe, predictable, and durable microbiota-based decolonization strategies.

### 5.3. Designer Strains with Kill Switches and Inducible Kynurenine Aminotransferase (KAT) Expression

Recent CRISPR-based kill switches in *E. coli* Nissle prove that dual chemical–temperature triggers can achieve >10^5^-fold clearance in the murine gut while retaining plasmid integrity over weeks—establishing a benchmark for biocontainment [29,229,230,231]. Parallel work in *Mycobacterium tuberculosis*, Bacillus Calmette–Guérin (BCG), and *Pseudomonas fluorescens* underscores two recurring concepts: circuit simplicity boosts genetic stability, and layered toxins curb escape [232]. Yet open questions persist. First, evolutionary pressure within complex microbiomes may favour cryptic recombination events; the long-read metagenomics of shed strains is needed to quantify real-world escape rates [233]. Second, few designs test function across variable pH, bile acids, or host temperatures; standardized “gut stress” challenge panels should precede human dosing [234]. Third, redundancy remains underused—stacking orthogonal CRISPRi, toxin–antitoxin, and auxotrophy modules could create multi-lock systems that tolerate single-node failure [235]. Fourth, kill switch burden on therapeutic payloads is rarely measured; metabolic toggle libraries and inducible promoters from xenogeneic silencing studies could minimize fitness costs [236]. Finally, regulatory pathways are hazy—agencies require validated shutoff diagnostics and environmental fate data [237]. The next steps include head-to-head comparisons of toxin cassettes, adaptive evolution assays in bioreactors using human stool, and digital polymerase chain reaction (PCR)-based field tests to monitor persistence post shedding [238]. Such work could convert kill switches from academic prototypes into deployable safeguards for LBPs.

### 5.4. Encapsulated “Post-Biotics” (e.g., Stabilized Kynurenic Acid (KYNA)) to Bypass Colonization

Skipped colonization formulations—enteric capsules, pH-responsive hydrogels, and microfluidic microparticles—now allow the direct delivery of stabilized KYNA and other post-biotics to the colon, sidestepping the variability of live engraftment [239]. Hydrogel and chitosan–alginate matrices protect labile metabolites from gastric acid, while spray-dried enteric microspheres and 3-D-printed capsules release cargo only at ≥pH 7, mirroring colonic transit [240]. Encapsulation reviews highlight that payload stability, release kinetics, and mucus penetration govern in vivo bioavailability, yet KYNA has never been loaded into these systems, and its solubility profile may demand excipient optimization [241]. Phage and enzyme encapsulation studies suggest that co-packaging metabolite pumps or β-glucuronidases could boost local concentration, but dose–response curves for post-biotic neuroprotection remain undefined [242]. The next steps include the following: screen generally recognized as safe (GRAS)-grade polymers for KYNA compatibility under accelerated aging; map release profiles in simulated gastrointestinal fluids and pig colonic explants; employ near-infrared-triggered nanocaps to test on-demand bursts during inflammation; and quantify systemic versus luminal KYNA using LC-MS in gnotobiotic mice, benchmarking against *Bifidobacterium*-produced levels [243]. Parallel human pilot studies can deploy encapsulated KYNA alongside wearable pH sensors to correlate release timing with mood and sleep metrics [244] (Table 6). Success in this regard would validate post-biotic capsules as a low-variance, regulatory-friendly alternative to live psychobiotics [245].

### 5.5. Cloud-Linked Stool Metabolomics Dashboards to Guide Weekly Probiotic Titration

Remote probiotic trials in COVID-19 and depression show patients can self-collect and courier fecal samples, while digital platforms already monitor calprotectin, zonulin, and secretory immunoglobulin A (sIgA) [246,247,248,249]. Yet strain-specific responses vary and optimal titration schedules remain guesswork [250]. Cloud-linked dashboards that stream LC-MS metabolite panels—SCFAs, indoles, and bile acid ratios—could provide weekly feedback loops to adjust probiotic dose or strain composition [251]. Key factors are as follows: multispecies probiotics shorten gut transit time and shift microbiota in constipation meta-analyses, but responses vary by baseline diet and Lactobacillus colonization predictors such as cheese and n-3 fatty acid intake; probiotics modulate clock gene expression and the gut–lung axis, implying time-of-day and symptom-phase windows for dosing; and large-scale genome scans map foodborne lactic acid bacteria that could lead to personalized consortia [246,252] (Table 5). Gaps include the lack of validated metabolite thresholds that signal “dose-too-low” versus “overshoot” issues and the absence of algorithms that incorporate diet, sleep, and medication logs alongside omics [253]. The next steps include building a reference library of weekly stool metabolomes from diverse cohorts on fixed probiotic regimens; training adaptive Bayesian models that recommend titration when variance-normalized SCFAs or indole scores drift beyond control limits; integrating wearable-captured feeding rhythms to schedule capsule timing; and running N-of-1 crossover trials to benchmark dashboard-guided titration against static dosing [254]. A successful system could transform probiotics from one-size-fits-all supplements into dynamic, biomarker-steered therapies [255] (Table 4).

## 6. Intervention 2.0: Dual Inhibitors, Exercise, and Real-Time Biosensing

Next-generation therapeutics are converging on a three-pronged strategy to disarm KYN metabolites in cancer and immune dysregulation [74,256,257]. First, dual inhibitors such as RY103 and ZC0101 simultaneously block IDO1 and TDO (or IDO1 and thioredoxin reductase), outperforming single-agent IDO1 blockade by collapsing redundant enzyme circuits and lowering intratumoral KYN to neuroprotective baselines [257,258]. Preclinical models show that these agents stunt glioma and pancreatic tumor growth by quenching KYN-AhR signaling and restoring cytotoxic T-cell traction [257,259]. Second, structured endurance exercise—a potent physiological lever—amplifies KYN re-balancing by accelerating peripheral KYN clearance via skeletal muscle KAT upregulation, creating a metabolic “sink” that synergizes with pharmacological inhibition [32,260]. Third, wearable biosensors now capture continuous lactate, glucose, and microvascular O_2_ data, while emerging implantable electrochemical probes detect real-time KYN-to-Try ratios in interstitial fluid [261]. Linking these feeds to adaptive dosing algorithms could personalize dual-inhibitor schedules around exercise bouts, maximizing metabolic windowing and minimizing toxicity [74,256,262]. Immediate next steps include human–machine interface trials that pair IDO1/TDO inhibitors with supervised exercise and real-time KYN monitoring, testing whether algorithm-guided titration enhances tumor response and rescues mood or fatigue [74,256,260,262]. “Intervention 2.0” fuses pharmacology, lifestyle, and biosensing into a closed-loop platform poised to redefine KYN-targeted precision medicine. Newly designed KYNA derivatives, refined by C-3 side-chain alterations, are being evaluated for their ability to fine-tune motor and cognitive outcomes [263,264].

### 6.1. Literature Review: Dual Inhibition and Kynurenine (KYN) Modulation

Early dual-inhibitor work centered on M4112, the first oral agent targeting both IDO1 and TDO [73,257,260,265]. Preclinically, M4112 halved tumor KYN/Trp ratios across xenograft panels; phase I results confirmed on-target plasma suppression with manageable fatigue and nausea, yet a compensatory rise in systemic KYN hinted at peripheral sinks that may blunt efficacy [73,260,265]. Parallel chemistry screens generated RY103 (IDO1/TDO) and ZC0101 (IDO1/TrxR), each extending the dual-hit concept to pancreatic and oxidative stress contexts, respectively [257,258]. These compounds curtailed migration, invasion, and colony formation in cell line models, supporting broad antitumor potential [257,258]. Outside cancer, pathway modulation remains complex. N-acetylcysteine attenuates cisplatin-induced cognitive decline by normalizing brain KYNA, whereas TNF-α blockade in rheumatoid arthritis leaves KYN flux largely untouched despite clinical remission, underscoring disease-specific regulation [266,267,268]. Human endotoxin challenge acutely spikes both neurotoxic QA and protective KYNA without correlating to sickness behavior, questioning causal links [269]. Observational cohorts add nuance: higher downstream metabolites (xanthurenic and picolinic acids) are associated with better survival and reduced fatigue in colorectal cancer survivors, yet KYN activation predicts mortality in acute respiratory distress syndrome [270,271]. Collectively, the literature depicts a heterogeneous KYN landscape where dual-enzyme blockade is promising but must contend with systemic metabolite reservoirs, context-dependent feedback loops, and variable host outcomes—parameters future trials must quantify.

### 6.2. Research Gaps: Adaptive Dose Timing and Real-Time Monitoring

Although adaptive designs are reshaping oncology and metabolic drug development, most trials still optimize dose magnitude while ignoring circadian or activity-linked timing [272]. Continuous glucose-monitoring data show that SGLT2 inhibitors lower glycemic variability yet raise ketoacidosis risk in type 1 diabetes, but no study dynamically shifts dosing around exercise or nocturnal hypoglycemia [273]. Therapeutic drug monitoring platforms for kinase inhibitors confirm exposure–toxicity windows, yet they do not integrate wearable-captured vital signs or KYN biomarkers to guide intraday titration [272,274]. Intermittent schedules for phosphoinositide 3-kinase (PI3K), Bruton’s tyrosine kinase (BTK), and Kirsten rat sarcoma viral oncogene homolog (KRAS) inhibitors reduce adverse events preclinically, but statistical frameworks treating “time-on” and “time-off” as co-randomized variables remain undeveloped [275,276]. Next-generation maturation and ropomyosin receptor kinase (TRK) inhibitors collect rich pharmacokinetic data but lack algorithms that pair those curves with real-time biosensor feeds [277,278]. Key gaps therefore include adaptive randomization models that incorporate dosing clock time as a modifiable arm; validated software bridges continuous glucose monitoring (CGM), lactate sensors, or KYN sensors and electronic trial master files; safety rules for rapid dose–time shifts in outpatient settings; and patient-reported outcome measures sensitive to circadian toxicity [279,280,281,282] (Table 4). Addressing these deficits will require multidisciplinary consortia linking chronobiologists, bioinformaticians, and trialists to pilot fully time-adaptive protocols for next-gen inhibitors and metabolic adjuvants.

### 6.3. Crosstalk Between Kynurenine (KYN) Pathway Modulation and Broader Metabolic Networks

The precision control of KYN metabolites, while promising, must be weighed against its biochemical entanglements. KYN metabolism is not isolated—it is woven into a dense network of metabolic systems, many of which are equally sensitive to perturbations. Modulating this pathway can create downstream effects, some beneficial, others potentially disruptive [283,284,285]. For example, inhibiting IDO1 and TDO may rebalance immunosuppressive Trp catabolism, but this can also constrict the NAD biosynthesis pipeline. Since NAD is central to redox reactions, mitochondrial respiration, and SIRT1 activity, its depletion may compromise energy metabolism in high-demand tissues such as muscle, gut, and immune organs [130,285]. At the same time, exercise-induced peripheral KAT upregulation shifts KYN toward KYNA, reducing neurotoxicity but also possibly influencing central glutamatergic tone, fat oxidation, and glycemic control [130,286]. Further complicating the landscape, KYN metabolites interact with the AhR, linking immune tone to lipid metabolism, insulin sensitivity, and barrier integrity. Feedback into circadian and epigenetic regulators via SIRT1-PGC1α loops suggests that KYN modulation could even entrain broader rhythmic and transcriptional programs [130,284,286]. Altogether, “Intervention 2.0” holds transformative potential—but not in a vacuum. Multiscale modeling, real-time flux profiling, and cross-pathway biomarker integration will be needed to map its full physiological footprint. Recognizing these interdependencies enables smarter and safer intervention designs and guards against tunnel vision in therapeutic innovation.

### 6.4. Phase-Ib “Smart Protocols”: Micro-Dosed Dual Inhibitors Guided by Saliva Kynurenic Acid (KYNA) Sensors

**Figure 6:**
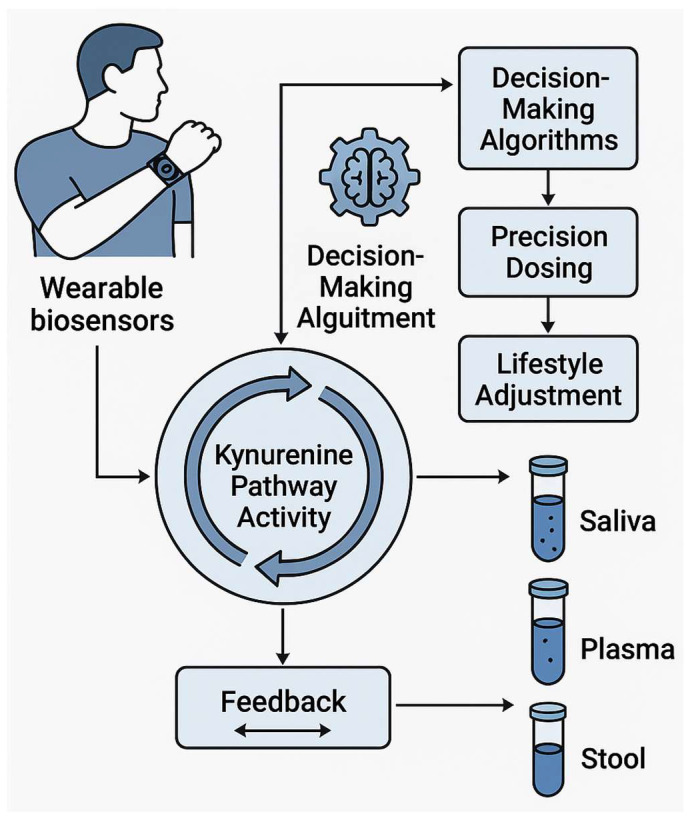
Adaptive intervention platform: Biomarker-guided smart protocols. Diagrammatic representation of closed-loop therapeutic framework (“Intervention 2.0”) integrating wearable biosensors, real-time biomarker readouts (saliva, plasma, stool), and artificial intelligence (AI)-driven decision-making algorithms. This model illustrates how precision dosing and lifestyle adjustments dynamically recalibrate kynurenine pathway activity.

Phase Ib studies already test dual phosphoinositide 3-kinase delta/gamma isoforms (PI3Kδ/γ) or spleen tyrosine kinase (SYK)/Fms-like tyrosine kinase 3 (FLT3) inhibitors with Bayesian dose-finding, yet none exploit non-invasive biosignatures to steer real-time titration [287]. Portable electrochemical strips now quantify KYNA in saliva within minutes and correlate with plasma levels, creating an opportunity for closed-loop dosing [288]. Conceptually, micro-doses of IDO1/TDO inhibitors could be administered every 6–8 h; if post-dose saliva KYNA falls below a personalized threshold, the algorithm triggers the next micro-dose, otherwise delays it—minimizing over-suppression and toxicity [289]. Thermal- and pH-responsive microspheres from dual-stimuli delivery research provide a vehicle for sub-gram, subcutaneous depots that release inhibitor pulses when externally warmed or after a salivary cue [290]. Trial design would integrate Bayesian optimal interval design for Phase 1/2 Trials (BOIN12) utility-based intervals, defining acceptable KYNA suppression windows rather than traditional maximum tolerated dose (MTD) [291]. Key gaps include validation of saliva–tumor KYNA concordance, stability of sensor reagents under recurrent sampling, and pharmacokinetic modeling of micro-pulse dynamics [292,293]. Immediate next steps are run a crossover pharmacokynetics study comparing saliva, plasma, and tumor microdialysate KYNA after RY103 micro-dosing; calibrate the Bayesian control algorithm using simulated patient data; and embed patient-reported fatigue and cognitive scores to test whether KYNA-targeted pacing improves tolerability relative to fixed bis in die (BID) regimens [294,295] (Table 6). Success would pioneer biomarker-responsive phase-Ib frameworks, setting a precedent for precision chronopharmacology (Figure 6).

### 6.5. Conceptual and Translational Limitations

While Intervention 2.0 presents a theoretically compelling synthesis of enzyme inhibition, behavioral modulation, and real-time monitoring, several fundamental limitations remain that temper its translational momentum.

First, many assumptions embedded in this framework hinge on linear causality—that manipulating one component (e.g., IDO1 activity) will predictably shift systemic outcomes. However, the KYN pathway is enmeshed in feedback-regulated networks influenced by age, sex, immunometabolic status, and microbial composition [32,130,286]. This makes “clean” cause–effect outcomes unlikely, particularly in heterogeneous clinical populations. Second, the real-time sensing of KYN metabolites, though conceptually exciting, remains technically underdeveloped. Unlike glucose or lactate, KYN intermediates are unstable, exist in low micromolar concentrations, and vary across biofluids. Translating high-resolution fluctuations into actionable feedback loops—without over-interpreting noise—is a major barrier to clinical deployment. Third, exercise as an intervention is notoriously context-dependent [32,130,296]. Its physiological impact on Trp-KYN flux may differ dramatically between pro-inflammatory and anabolic states, across circadian phases, or based on training history. Designing standardized, reproducible exercise prescriptions that reliably modulate KAT/IDOs dynamics across patients will be inherently difficult. Finally, the pharmacological inhibition of IDO1/TDO may introduce off-target effects, especially in tissues dependent on NAD salvage pathways or those with high basal immune turnover [285,286,297]. The long-term consequences of redirecting tryptophan away from the KYN metabolic pathway—particularly in vulnerable systems like the CNS—remain underexplored [297,298].

Thus, while the “what if” scenarios are rich in mechanistic possibility, the “how” remains constrained by technical, biological, and regulatory bottlenecks. Addressing these barriers will require multi-disciplinary co-design involving molecular biologists, bioengineers, exercise physiologists, and clinical trialists alike.

### 6.6. Artificial Intelligence (AI)-Driven Feedback Loops That Auto-Adjust Evening Treadmill Sessions or Probiotic Cocktails Based on Morning Kynurenine (KYN)/Tryptophan (Trp) Slope: AI-Driven KYN/Trp Feedback Loops

Emerging hardware and informatics can now pair morning finger-stick KYN-to-Trp slopes with adaptive lifestyle prescriptions that update nightly [283,299,300,301]. AI-controlled treadmills and exoskeletons already modulate belt speed or torque in real time from gait and heart rate inputs; integrating cloud-fed metabolite data would allow the algorithm to lengthen or intensify an evening run only when the day’s KYN/Trp ratio signals pro-inflammatory drift [32,130,132,300]. Likewise, modular probiotic cocktails that shift gut indole and short-chain fatty acid production could be titrated each afternoon, with doses nudged up when the biomarker slope exceeds a personalized threshold [47,300,301,302]. Clues supporting feasibility include the following: clinical artificial intelligence operation (ClinAIOp) frameworks for continuous therapeutic monitoring in hypertension and diabetes; Kinect- or sensor-driven treadmills that already auto-pace speed by user position; and murine and human studies where tailored probiotic blends reduce intestinal inflammation and modulate Trp metabolism [32,38,301,302] (Table 6). Gaps remain. No longitudinal dataset links daily KYN/Trp excursions with exercise intensity or probiotic-induced metabolomic shifts. Sensor validation for dried-blood-spot or saliva Trp/KYN needs real-world robustness, and reinforcement learning models must balance metabolic targets against user fatigue and adherence [32,283,299,300]. The next steps: deploy a 12-week N-of-1 crossover where participants collect morning KYN/Trp, receive algorithm-set treadmill or probiotic adjustments, and stream compliance plus mood, glucose, and heart rate metrics. Success in this regard would help create a prototype of closed-loop “exercise–microbiome” medicine that personalizes both movement and microbes to biochemical feedback.

## 7. Conclusions

Harnessing the gut–KYN axis demands an integrative framework that unites microbiology, neuro-immunology, and chronomedicine. This review demonstrates how spatial “checkpoints,” circadian and sex modifiers, and engineered microbiota collectively steer Trp’s fate toward either neuroprotection or pathology. By weaving enzymatic dual inhibition with lifestyle levers—exercise, diet, and real-time biosensing—the authors chart a precision medicine roadmap that transcends siloed approaches. Theoretical insight lies in reframing the KYN metabolism as a dynamically gated network rather than a linear cascade; practical value emerges in the proposed closed-loop trials that titrate inhibitors, probiotics, or treadmill load to biomarker feedback. Yet critical questions remain: Which cell-specific KYN fluxes truly drive disease? How stable are engineered consortia in the complex gut ecosystem? What statistical architectures best randomize dosing time as well as dose? Addressing these gaps will require single-cell multi-omics, longitudinal metabolite dashboards, and adaptive, sex-balanced clinical designs. These advances are underpinned by AI integration and the development of human-specific platforms, which promise to transform the personalization of neuropsychiatric care [303,304]. Advancing these fronts will not only refine KYN-targeted therapeutics but also provide a template for biomarker-guided interventions across metabolic and neuroimmune disorders.

## Figures and Tables

**Table 1:** Three translational frontiers in tryptophan (Trp)–kynurenine (KYN) research.

	Category	Description/Core Issue	Implication/Goal
Translational Challenge			
1.	Causal Mapping	Thousands of disease associations but no causal framework linking microbiota, host enzymes (IDO1, TDO) and downstream metabolites (KYNA, QA) to physiology	Limits precision design of probiotics, enzyme inhibitors, lifestyle prescriptions
2.	Spatial Resolution	Bulk assays mask cell- and tissue-specific “checkpoints” (astrocytes, microglia, BBB endothelium)	Demands targeted modulation of localized hotspots rather than pathway-wide blockade
3.	Temporal Dynamics	Trp–KYN flux oscillates with circadian rhythms and sex hormones; chronotherapeutic windows under-studied	Missing optimal timing may blunt efficacy or raise toxicity of interventions
Key Objective			
1.	Map Spatial Checkpoints	Chart localized KYN metabolism niches in brain and periphery	Inform cell-type-specific therapeutic targeting
2.	Characterize Sex and Circadian Modifiers	Define how hormones and clocks tilt Trp metabolism toward neurotoxicity or resilience	Enable time- and sex-specific dosing strategies
3.	Develop Microbiota-Based Precision Switches	Engineer probiotic consortia and post-biotics that reroute Trp flux	Provide modular, patient-tailored metabolic control
4.	Outline Integrated “Intervention 2.0” Platform	Combine dual-enzyme inhibitors, exercise, and AI-driven biosensing	Create closed-loop, adaptive therapeutics

BBB, blood–brain barrier; IDO1, indoleamine 2,3-dioxygenase 1; KYN, kynurenine; KYNA, kynurenic acid; QA, quinolinic acid; TDO, tryptophan 2,3-dioxygenase; Trp, tryptophan.

**Table 2:** Precision modulation of neurovascular kynurenine (KYN) metabolism.

Experimental Strategy	Mechanistic Lever/Tools	Mechanistic Lever/Tools	Expected Outcome/ Advantage
Endothelial CRISPRi “zip-code” AAV-targeted knock-down of KMO or KYNU in perivascular endothelium	ZIM3-KRAB CRISPRi cassette Endothelial-specific promoters + miRNA “clearing tags” Bar-coded AAV libraries	Build bar-coded AAV panels to sharpen endothelial specificity Validate knock-down and KYN–metabolite flux in brain slice co-cultures Monitor glutamate dynamics in vivo with optogenetic reporters Test impact on tumor infiltration and behavior (KMO-high metastasis models) Run parallel safety screens for NAD pools and mitochondrial stress	Precise, vessel-restricted suppression of 3-HK/QA; dampened excitotoxicity and immune escape with minimal systemic off-target effects
Light-addressable riboswitch control in astrocytes in milliseconds, reversible tuning of KMO/KYNU translation	Photocleavable or Z-lock riboswitch fused to target mRNA Pulsed IR/visible light for on–off gating Simultaneous GCaMP or glutamate sensor read-outs	Package riboswitch construct in astrocyte-specific AAV Benchmark translation kinetics vs. Ca^2+^ rise in organotypic slices Map spatial spread of KYN pulses and gliotransmitter waves Deploy fiber-coupled two-photon uncaging in vivo to test network excitability during sleep, seizure, learning	Real-time, non-invasive “dimmer switch” for KP activity with built-in metabolic read-outs; ideal for dissecting causal links between KYN flux and neural circuitry

AAV, adeno-associated virus; CRISPRi, clustered regularly interspaced short palindromic repeats interference; GCaMP, green fluorescent protein–calmodulin–M13 peptide fusion; 3-HK, 3-hydroxykynurenine; IR, infrared; KMO, kynurenine 3-monooxygenase; KP, kynurenine pathway; KYN, kynurenine; KYNU, kynureninase; mRNA, messenger ribonucleic acid; miRNA, micro ribonucleic acid; NAD, nicotinamide adenine dinucleotide; QA, quinolinic acid; ZIM3, zinc finger protein 3; KRAB, Krüppel-associated box.

**Table 3:** Chronobiological influences and biomarker-guided intervention strategies.

Circadian/Sex-Specific Gap	Why It Matters	Biomarker-Guided Next Step	Anticipated Pay-Off
Absence of chronopharmacology trials for IDO1/TDO, KMO or KAT inhibitors	Optimal dosing windows are unknown; schedules may blunt efficacy or raise toxicity	Launch Bayesian adaptive trials that co-randomize dose and clock time, using real-time KYN/QA read-outs as decision boundaries	Evidence-based chrono-dosing algorithms, reduced off-target effects
Inadequate stratification by circadian phase and sex	Female-specific PK/PD and toxicity signals vanish when averaged	Embed wearable-derived chronotype + hormonal phase into inclusion criteria; pre-specify sex-by-time interaction models	Sex-aware precision medicine; higher treatment tolerability
Undefined mechanistic links between clock genes, hormones and KYN enzyme activity	Surrogate biomarkers risk misinterpretation without pathway context	Overlay 24 h cortisol/melatonin rhythms onto multi-time-point KYN, QA, KYNA panels; apply mixed-effects chronobiology models	Mechanistic targets for combination therapy; validated biomarkers
Wearable metrics (light, sleep) not integrated into study design	Zeitgebers that modulate KYN flux are ignored	Trigger capillary micro-sampling when lux-derived phase-angle deviation crosses threshold (“biomarker-in-the-loop”)	Personalized sampling and dosing windows; lower noise in endpoints
Sex- and light-cycle biases in pre-clinical models	Male-only, fixed-light studies limit translation	Use sex-balanced rodents under rotating light cycles; validate with humanized microbiome models	Higher translational validity of pre-clinical findings
Lack of validated rapid biomarkers to couple KYN swings to outcomes	Real-time dose adjustment impossible	Develop saliva/finger-stick electrochemical strips for KYN/Trp/QA; calibrate against plasma and microdialysate	Closed-loop dose titration; faster early-phase trials
CM Focus: QA spikes during night-shift work	Neurotoxic burden may rise, especially in vulnerable chron otypes	Pilot cross-over study: shift workers + hourly capillary sampling + light and activity trackers Model QA vs. lux-derived phase angle (mixed-effects) Overlay cortisol and melatonin to disentangle stress vs. circadian drivers Test timed blue-light blockers, melatonin, or time-restricted feeding	Identifies high-risk chronotypes and intervention windows; informs occupational health policies

CM, circadian misalignment; IDO1, indoleamine 2,3-dioxygenase 1; KAT, kynurenine aminotransferase; KMO, kynurenine 3-monooxygenase; KYN, kynurenine; KYNA, kynurenic acid; PD, pharmacodynamic drug effect and mechanism; PK, drug absorption, distribution, metabolism, excretion; QA, quinolinic acid; TDO, tryptophan 2,3-dioxygenase; Trp, tryptophan.

**Table 4:** Next steps for adaptive chronopharmacology and dosing optimization.

Adaptive Gap/ Focus Area	Actionable Strategy and Tool Kit	Key Operational Step(s)	Intended Pay-Off
Dose–Time Randomization	Bayesian hierarchical designs that co-randomize dose level + clock time	Simulate designs borrowing strength across adjacent time bins Integrate wearable-derived chronotype into priors Embed rolling interim analyses that reduce unfavorable time windows rather than doses	Evidence-based chrono-dosing rules; smaller, faster trials
Sensor–Data Pipeline	Validated software bridges from CGM/lactate/KYN sensors and electronic TMF	Build real-time API between biosensors and trial master file Version-control data streams for audit compliance	Seamless biomarker ingestion; regulatory-ready data fidelity
Biomarker Validation	Rapid KYN/Trp/QA saliva or finger-stick electrochemical strips	Cross-validate saliva, plasma, tumor microdialysate after micro-dosed dual-inhibitor (e.g., RY103) crossover PK study	Closed-loop dosing feasible at point of care
Safety Governance	Rules for rapid dose–time shifts in outpatient settings	Use melatonin/cortisol point-of-care assays as safety triggers Pre-define algorithmic “pause” thresholds	Protects patients while enabling flexible chrono-titration
Patient-Centric Metrics	PROMs tuned to circadian toxicity (fatigue, cognition)	Embed in ePRO platform; couple to Bayesian controller as soft constraints	Holistic tolerability; improves adherence
Pilot Implementation	First-in-human chrono-trials for drugs with known chronotoxicities	Pilot crossover study: micro-dosed dual IDO1/TDO inhibitor + real-time KYNA pacing Roll out in oncology and mood disorder cohorts	Proof-of-concept that algorithm-guided timing beats fixed BID regimens

API, application programming interface; BID, twice-daily; CGM, continuous glucose monitoring; IDO1, indoleamine 2,3-dioxygenase 1; ePRO, electronic patient-reported outcomes; KYN, kynurenine; KYNA, kynurenic acid; PK, pharmacokinetic; PROMs, patient-reported outcome measures; QA, quinolinic acid; TDO, tryptophan 2,3-dioxygenase; TMF, trial master file; Trp, tryptophan.

**Table 5:** Precision microbiota-based therapeutics.

Development Challenge/Key Insight		Why It Matters	Precision Strategy or Next Step
1.	Unpredictable engraftment and variable response Engineered consortia (e.g., VE303) often need antibiotic conditioning. Multispecies probiotics cut transit time only in diet-matched responders.	Long-term decolonization or mood relief can fade.	- Screen baseline diet and Lactobacillus/Bifidobacterium colonization predictors before enrollment. - Tailor consortia composition to individual fermentable fiber intake.
2.	Scarce durability and mechanistic data Limited longitudinal sequencing of phage–bacteria–host dynamics.	Regulatory roadblock; patient safety.	- Pair shotgun metagenomics with phage omics in 6–12-month follow-up. - Validate causal metabolites via gnotobiotic “plug-and-play” models.
3.	Safety and HGT risks CRISPR-edited *E. coli* microcin strains could acquire resistance cassettes.	Batch-to-batch variability in metabolite output undermines reproducibility.	- Stack orthogonal kill switches (CRISPRi + toxin–antitoxin + auxotrophy). - Deploy long-read sequencing of shed strains to monitor HGT.
4.	Manufacturing and QC deficits.	Missing optimal dosing windows weakens efficacy.	- Adopt GMP-aligned metabolite “fingerprinting” for every lot. - Introduce in-line mass spec release tests.
5.	Timing factors ignored Circadian rhythms gate colonization resistance; probiotics modulate clock gene expression.	Jurisdictional differences delay global rollout.	- Run adaptive trials that modulate dosing relative to light exposure, meals, and antibiotics. - Schedule capsule release (enteric or pH-triggered) to late-day circadian troughs in QA.
6.	Fragmented regulatory pathways.	One-size-fits-all combinations underperform.	- Establish harmonized guidance for genetically modified live biotherapeutic products (LBPs) via international consortia.
7.	Personalized strain selection Foodborne lactic acid bacteria genomes map “starter” strains for personalized consortia.	Long-term decolonization or mood relief can fade.	- Build a strain library ranked by individual dietary patterns and metabolite deficits; auto-compose patient-specific mixes.

CRISPR, clustered regularly interspaced short palindromic repeats; CRISPRi, clustered regularly interspaced short palindromic repeats interference; GMP, good manufacturing practice; HGT, horizontal-gene-transfer; QA, quinolinic acid; QC, quality control.

**Table 6:** Advanced delivery systems and artificial intelligence (AI)-driven adaptive therapeutics.

Innovation Track	Key Development Step	Implementation Path/Intended Pay-Off
Encapsulated Post-Biotics (e.g., KYNA)	▸ Screen GRAS-grade polymers and hydrogels for KYNA stability under accelerated aging. ▸ Map release profiles in simulated GI fluids and pig colonic explants. ▸ Test near-infrared-triggered nanocapsules for on-demand bursts during inflammation. ▸ Quantify systemic vs. luminal KYNA via LC-MS in gnotobiotic mice, benchmark against Bifidobacterium output.	Locks in metabolite potency until it reaches the colon. Enables flare-responsive “burst” therapy. Provides pharmacokinetic data to support dosing equivalence to live microbe production.
Adaptive Probiotic Titration	▸ Build a reference library of weekly stool metabolomes from diverse cohorts on fixed probiotic regimens. ▸ Train adaptive Bayesian models that recommend dose or strain tweaks when SCFA/indole scores drift. ▸ Integrate wearable-captured feeding rhythms to optimize capsule timing. ▸ Run N-of-1 cross-over trials comparing dashboard-guided vs. static dosing.	Converts one-size-fits-all probiotics into dynamic, biomarker-steered therapies. Aligns delivery with each person’s meal schedule and gut motility. Generates individualized responder fingerprints for precision formulation.
AI-Driven Gut–Brain Feedback Loops	▸ Leverage ClinAIOp frameworks for continuous therapeutic monitoring (glucose, lactate, KYN sensors). ▸ Fuse real-time metabolite slopes with sensor-driven exercise platforms (e.g., auto-pacing treadmills). ▸ Embed adaptive probiotic dashboards into the same loop for daily strain/dose updates.	Closed-loop system that tweaks movement and microbes to keep KYN/Trp in a protective range. Minimizes clinician workload—algorithm adjusts interventions overnight. Sets the stage for fully autonomous “gut–brain wearables” in neuropsychiatric care.

AI, artificial intelligence; ClinAIOp, clinical artificial intelligence operations; GI, gastrointestinal; GRAS, generally recognized as safe, KYN, kynurenine; KYNA, kynurenic acid; LC-MS, liquid chromatography–mass spectrometry; N-of-1, single-patient; SCFA, short-chain fatty acid; Trp, tryptophan.

## Abbreviations

The following abbreviations are used in this manuscript:

AAV	adeno-associated virus
AD	Alzheimer’s disease
AhR	ary hydrocarbon receptor
AI	artificial intelligence
BBB	blood–brain barrier
CM	circadian misalignment
COVID-19	coronavirus disease 2019
CRISPR	clustered regularly interspaced short palindromic repeats
CRISPRi	clustered regularly interspaced short palindromic repeats interference
IDO1	indoleamine 2,3-dioxygenase 1
KMO	kynurenine 3-monooxygenase
KYN	kynurenine
KYNU	kynureninase
KYNA	kynurenic acid
KAT	kynurenine aminotransferase
LBPs	live biotherapeutic products
LC-MS	liquid chromatography–mass spectrometry
NAD	nicotinamide adenine dinucleotide
QA	quinolinic acid
SCFAs	short-chain fatty acids
TDO	tryptophan 2,3-dioxygenase
TLR	Toll-like receptor
Trp	tryptophan
ZIM3	zinc finger protein 3
